# Skeletal muscle metabolic adaptations to endurance exercise training are attainable in mice with simvastatin treatment

**DOI:** 10.1371/journal.pone.0172551

**Published:** 2017-02-16

**Authors:** William M. Southern, Anna S. Nichenko, Daniel D. Shill, Corey C. Spencer, Nathan T. Jenkins, Kevin K. McCully, Jarrod A. Call

**Affiliations:** 1 Department of Kinesiology, University of Georgia, Athens, Georgia, United States of America; 2 Regenerative Bioscience Center, University of Georgia, Athens, Georgia, United States of America; Universidad Europea de Madrid, SPAIN

## Abstract

We tested the hypothesis that a 6-week regimen of simvastatin would attenuate skeletal muscle adaptation to low-intensity exercise. Male C57BL/6J wildtype mice were subjected to 6-weeks of voluntary wheel running or normal cage activities with or without simvastatin treatment (20 mg/kg/d, n = 7–8 per group). Adaptations in *in vivo* fatigue resistance were determined by a treadmill running test, and by ankle plantarflexor contractile assessment. The tibialis anterior, gastrocnemius, and plantaris muscles were evaluated for exercised-induced mitochondrial adaptations (i.e., biogenesis, function, autophagy). There was no difference in weekly wheel running distance between control and simvastatin-treated mice (P = 0.51). Trained mice had greater treadmill running distance (296%, *P*<0.001), and ankle plantarflexor contractile fatigue resistance (9%, *P*<0.05) compared to sedentary mice, independent of simvastatin treatment. At the cellular level, trained mice had greater mitochondrial biogenesis (e.g., ~2-fold greater *PGC1α* expression, *P*<0.05) and mitochondrial content (e.g., 25% greater citrate synthase activity, *P*<0.05), independent of simvastatin treatment. Mitochondrial autophagy-related protein contents were greater in trained mice (e.g., 40% greater Bnip3, *P*<0.05), independent of simvastatin treatment. However, Drp1, a marker of mitochondrial fission, was less in simvastatin treated mice, independent of exercise training, and there was a significant interaction between training and statin treatment (*P*<0.022) for LC3-II protein content, a marker of autophagy flux. These data indicate that whole body and skeletal muscle adaptations to endurance exercise training are attainable with simvastatin treatment, but simvastatin may have side effects on muscle mitochondrial maintenance via autophagy, which could have long-term implications on muscle health.

## Introduction

Cardiovascular disease (CVD) is the leading cause of death in the United States, with an estimated 1 in 3 adults presenting the disease [1]. Statin drugs are the most prescribed and effective pharmacological therapy for the reduction of CVD-related mortality [2]. The mechanism of action of statins is the competitive inhibition of 3-hydroxy-3methylglutaryl coenzyme A reductase, the rate-limiting enzyme in the cholesterol biosynthesis pathway. Reduced cholesterol biosynthesis leads to a decline in circulating LDL levels that contributes to the reduced risk of CVD mortality. Regular exercise is also recognized as an effective means to reduce CVD mortality risk [3] and individuals seeking to reduce their risk of CVD may implement both statin therapy and exercise simultaneously. Statin use has been linked with skeletal muscle myopathies such as cramps, soreness, stiffness, and weakness [4], although direct evidence of skeletal muscle dysfunction is limited and inconclusive. Specifically, few studies have objectively tested skeletal muscle contractile function with statin use, and several of the studies that have report no adverse impact of statin use on skeletal muscle contractility [5–10]. Moreover, only an estimated 9–13% of statin users report statin-induced muscle cramps, soreness, or stiffness [4, 10].

There is also limited evidence suggesting that statins adversely affect exercise capacity [11, 12] and exercise training-induced cardiovascular and skeletal muscle adaptations [13]. A proposed cause of statin-related myopathies is skeletal muscle mitochondrial dysfunction [12, 14–16], which could potentially give rise to statin-induced attenuation of skeletal muscle exercise training adaptions. However, the effect of statin treatment on cardiovascular or skeletal muscle exercise training-induced adaptations remains unclear. For this investigation, we were particularly interested in testing if acute exercise adaptations were possible prior to a potential accumulation of contraindicative effects of statin use. Therefore, the purpose of this study was to evaluate the extent to which statin treatment altered exercise capacity and skeletal muscle adaptations to endurance exercise training. We hypothesized that exercise capacity and skeletal muscle exercise adaptations to endurance exercise would be impaired with statin treatment.

## Materials and methods

### Ethics statement

All protocols were approved by the University of Georgia Institutional Animal Care and Use Committee (A2014 08-031-Y2-A5). All efforts were made to minimize animal suffering.

### Animals and study design

Male C57BL/6J mice aged 8 wk were purchased from Jackson Laboratories and housed at 20–23°C on a 12:12 hour light:dark cycle with food and water *ad libitum*. Mice were randomly assigned to one of four conditions, sedentary (*n* = 8), sedentary with simvastatin treatment (*n* = 7), wheel running (*n* = 8), and wheel running with simvastatin treatment (*n* = 8). Sample size calculations were based on *PGC-1α* mRNA expression as it is a robust indicator of mitochondrial biogenesis [17]. A sample size of at least 5 per group was calculated *a priori* as sufficient to detect a 2-fold increase in *PGC-1α* mRNA expression assuming a power of 0.8 and a α-level of 0.05. All mice were assessed for treadmill running aptitude following the 6-week study, and 72-hours after the treadmill running test mice were assessed for ankle plantarflexion muscle function *in vivo*. Gastrocnemius, plantaris, soleus, tibialis anterior, and extensor digitorum longus muscle were dissected, weighed, and either immediately processed for tissue analysis or snap frozen in liquid nitrogen and stored at -80°C. While under anesthesia, the mice were then sacrificed by excision of the heart.

This study was carried out in strict accordance with the recommendations in the Guide for the Care and Use of Laboratory Animals of the National Institutes of Health.

### Simvastatin treatment and wheel running

Simvastatin was used for this investigation because it has a well-established link to adverse effects of skeletal muscle mitochondrial [13, 16, 18] and is currently the most prescribed statin drug in the U.S. [19]. We chose administer a simvastatin dose of 20 mg/kg/day through the drinking water. This dose corresponds to a human equivalent dose of ~100 mg/day for a 60 kg adult [20], which is just above the highest approved simvastatin dose of 80 mg/day. Simvastatin powder (Sigma-Aldrich) is naturally insoluble in water and therefore was initially dissolved in DMSO prior to mixing with drinking water as described by others [21]. Control mice received DMSO/water at the same concentration as the statin/DMSO/water mix. For all groups, water was replaced every other day, and consumption was monitored by collecting water bottle mass before and after bottle replacement. The 6-week statin treatment duration of this study corresponds to approximately 4.6 human years for mature adult mice between the age of 3 to 8 months [22]

Each trained mouse was housed individually and given free access to a running wheel (Columbus Instruments, Columbus, Ohio), while sedentary mice were housed in a standard mouse cage without access to a running wheel. Daily running totals were calculated from wheel revolutions collected at 5 min intervals. Mice were excluded from the entire dataset if they did not average at least 1500 m/day for any given week during the course of the study (n = 2 from run+DMSO treatment group) [23]. Additional mice that met the weekly running criteria were added to the study to replace excluded mice.

### Treadmill running

Each mouse was familiarized with the treadmill for five minutes at low speeds (2–5 m/min) for three consecutive days prior to the treadmill testing. On the day of the test, resting lactate was measured (Arkray Inc., Kyoto, Japan) via tail-snip and mice were placed on the treadmill (Columbus Instruments, Columbus, Ohio) in individual lanes. The treadmill protocol started with two stages, 7.5 m/min for 7 min and 10 m/min for 7 min followed by an increase of 2.5 m/min every 10 min until a maximum speed was achieved of 27.5 m/min [24, 25]. Percent grade remained constant throughout the test at 5%. Brushes at the end of the treadmill lane encouraged mice to keep running throughout the test. The fatigue test was terminated when mice no longer responded to 5 consecutive, forceful taps with the brushes. Upon fatigue, mice were quickly removed from treadmill lane and a post-test lactate reading was obtained. Treadmill distance and time were recorded for all mice.

### *In vivo* muscle function

*In vivo* peak isometric torque of the ankle plantarflexors was assessed as previously described [26]. Briefly, anesthesia was induced using an induction chamber and 5% isoflurane in oxygen. Anesthesia was maintained using 1.5% isoflurane at an oxygen flow rate of 0.4L/min. The left hindlimb was depilated and aseptically prepared and the foot placed in a foot-plate attached to a servomotor (Model 300C-LR; Aurora Scientific, Aurora, Ontario, Canada). The left peroneal nerve was severed and platinum-iridium needle electrodes (Model E2-12; Grass Technologies, West Warwick, RI) were placed on either side of the sciatic nerve to elicit contraction of the plantarflexor muscles [27]. Peak isometric torque was defined as the greatest torque measured during a 200-ms stimulation using 1-ms square-wave pulses at 300 Hz and increasing amperage 0.6 to 2.0 mA (models S48 and SIU5; Grass Technologies). Fatigability of the plantarflexor muscles was assessed using a protocol that has been shown to induce ~50% torque loss over the course of 120 contractions by using physiological stimulation frequencies of the hind limb, which range from 45–60 Hz [28, 29]. Briefly, for this study, the mice performed 120 submaximal isometric contractions for 2 min using 330 ms stimulations at 50 Hz.

### Gene expression

cDNA was generated from isolated gastrocnemius muscle RNA using a High Capacity cDNA Reverse Transcription Kit (Applied Biosystems, Foster City, CA). iQ SYBR Green Supermix (Bio-Rad) and sequence-specific primers were used to assess mRNA levels for the following genes, *PGC-1α*, (For: 5’-AGC CGT GAC CAC TGA CAA CGA G-3’; Rev: 5’-GCT GCA TGG TTC TGA GTG CTA AG-3’) *COXIV* (For: 5’-GCC TTG GAC GGC GGA AT-3’; Rev: 5’-CCA CAT CAG GCA AGG GGT AG-3’), *NOS3* (For: 5’-GCA TGG GCA ACT TGA AGA GTG-3’; Rev: CTT GCC GCA CAG CCC TAA AC-3’), *CD31* (For: 5’-AGC CAA CAG CCA TTA CGG TTA-3’; Rev: 5’-AGC CTT CCG TTC TCT TGG TG-3’), and *CDH5* (For: 5’-CCT GAG GCA ATC AAC TGT GC-3; Rev: 5’-GGA GGA GCT GAT CTT GTC CG-3’). Gene expression analysis was carried out by qRT-PCR on a Bio-Rad CFX instrument as previously described [30]. Data were normalized to 18S (For: 5’-TTG ATT AAG TCC CTG CCC TTT GT-3’; Rev: 5’-CGA TCC GAG GGC CTA ACT A-3’) and relative gene expression was calculated using the 2^-ΔΔCT^ method.

### Mitochondrial assays

Gastrocnemius muscles were homogenized in a glass tissue grinder in 33mM phosphate buffer (pH = 7.0) at a muscle:buffer ratio of 1:20. Citrate synthase (CS), β-hydroxy acyl-CoA dehydrogenase (β-HAD) activities were determined in triplicate as previously described [31]. Enzyme activities were normalized to total protein content (BCA; Thermo Fisher Scientific, Grand Island, New York).

For isolated mitochondrial respiration, mitochondria from tibialis anterior muscles were isolated and assessed for state 4, state 3, and state 3 uncoupled oxygen consumption according to the protocol outlined by Garcia-Cazarin et al. [32]. Briefly, mitochondria were isolated using a series of centrifugation steps performed at 0–4°C and resuspended in a final 100 μL of isolation buffer [32]. Protein concentration of the mitochondrial homogenate was determined via BCA assay. All measurements were performed using a Clark-type electrode (Oxygraph Plus System, Hansatech Instruments, UK) at 25°C. Prior to each experiment, the electrode was calibrated according to the manufacturer’s instructions and 500 μl of experimental buffer [32] was added to the chamber. 100 μg of isolated mitochondrial were loaded into the Oxygraph chamber. State 4 respiration (leak respiration in the absence of ADP) was initiated by the addition of 10 μL of 250 mM/125 mM glutamate/malate. State 3 respiration (respiration coupled to ATP synthesis) was initiated by the addition of 2 × 10 μL of 10 mM ADP/Succinate. State 3 uncoupled respiration (respiration uncoupled from ATP synthesis) was initiated by the addition of 0.5 μL of 0.1 mM FCCP, and cyanide (2 μL of 250 mM) was added last to terminate mitochondrial respiration. The respiratory control ratio (RCR) was defined as state 3 divided by state 4 respiration. An RCR of ≥ 4 was considered a viable mitochondrial preparation [32]. To account for differences in mitochondrial content between isolated mitochondrial fractions, all respiration rates were normalized by citrate synthase activities for each fraction.

### Autophagy-related protein content

For protein content analysis, 30 μg of total protein from each plantaris muscle was separated by SDS-PAGE, transferred onto a PVDF membrane, and immunoblotted as previously described [33]. The following antibodies (Cell Signaling, Danvers, MA) were used: Ulk1 (1:1000), phospho-Ulk1 (1:1000), Drp1 (1:1000), beclin-1 (1:1000), SQSTM1/p62 (1:1000), Bnip3 (1:1000), LC3B (1:1000), and mitofusin-2 (1:1000). Membranes were analyzed and quantified using Bio-Rad Laboratories Image Lab software (Hercules, CA).

### Statistical analyses

Data are presented in the results as mean ± SD. A multi-factor repeated measures analysis of variance (ANOVA) was used to analyze body mass, daily water consumption, or wheel running performance. The between-subject factors were simvastatin treatment and wheel running and the repeated measure factor was time. Two-way ANOVA was used to analyze the remaining data. All data were required to pass normality (Shapiro-Wilk) and equal variance tests (Brown-Forsythe *F* test) before proceeding with the ANOVA. Differences among groups are only reported where significant interactions were observed and subsequently tested with Tukey’s *post hoc* test using JMP statistical software (SAS, Cary, NC). Group main effects are reported where significant interactions were not observed. An α level of 0.05 was used for all analyses.

## Results

### Simvastatin treatment and wheel running

To monitor the dose of simvastatin the mice received throughout the course of the study, drinking water consumption was recorded daily. Daily water intake was ~44.8% higher among trained mice compared to sedentary mice (main effect: *P* < 0.001), independent of statin treatment. Given the daily water consumption and the concentration of simvastatin in the water, sedentary simvastatin treated mice received an estimated dose of 22.1 ± 2.8 mg/kg/day and the trained simvastatin treated mice received 34.8 ± 5.4 mg/kg/day. Throughout the study, body mass was recorded weekly and significantly increased ~12% from baseline independent of simvastatin treatment and exercise training (time effect: *P* <0.0001, baseline: 24.4 ± 1.4 g; week 6: 27.3 ± 1.5 g). Voluntary wheel running distance was recorded daily to determine if both control and simvastatin treated mice were running to the same extent and thus receiving a similar exercise stimulus. Both control and simvastatin mice had an equivalent training stimulus, as no differences were found in average weekly running distance (m/24 h) (*P* = 0.51), and trained mice averaged ~5.3 km per day of voluntary wheel running across the 6-week study.

### Treadmill running

**Fig 1:**
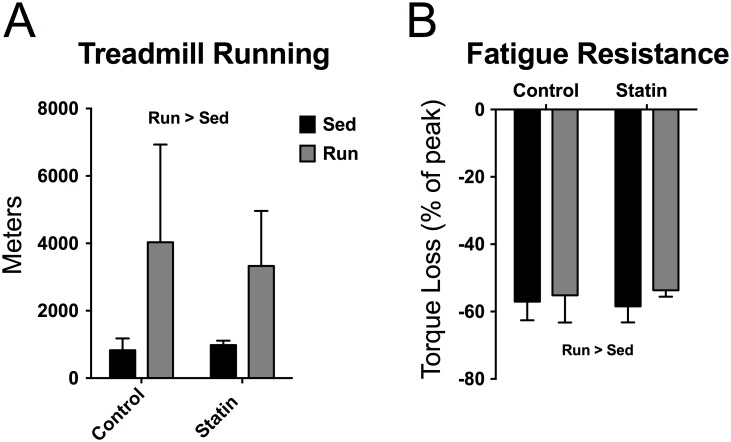
The effects of exercise training and simvastatin treatment on treadmill running capacity and contractile fatigue resistance (n = 7–8 per exercise/treatment group). *A*: Distance run during a progressive velocity treadmill running test to exhaustion. Run > Sed indicates main effect of training, *P* < 0.001. *B*: Plantarflexor torque loss following a fatiguing bout of 120 contractions. Run > Sed indicates main effect of training, *P* < 0.040. Torque loss is expressed as a percent of the peak torque generated during the protocol.

We started our systematic evaluation by first testing if simvastatin treatment affected fatigue resistance during an acute bout of intense exercise. During the treadmill running test, trained mice ran ~296% more meters than sedentary mice independent of simvastatin treatment (*P* < 0.001, Fig 1A). Blood lactate changes were used to ensure all mice, sedentary and trained, ran until exhaustion. Immediately following the treadmill running test, all groups had elevated blood lactate (~1.7 mmol/L increase from baseline; time effect: *P* < 0.001) independent of treatment and exercise groups. Treadmill running test performance is a measure of cardiorespiratory exercise adaptations as well as an indirect measurement of skeletal muscle exercise adaptations. To directly test the impact of simvastatin treatment on skeletal muscle contractile adaptations to endurance exercise training, we next performed *in vivo* contractile assessment of the ankle plantarflexors.

### *In vivo* muscle function

Trained mice had significantly less plantarflexion torque loss during a metabolically challenging contractile test compared to sedentary mice, independent of simvastatin treatment (*P* = 0.040, Fig 1B). By the end of the fatiguing protocol, trained mice only lost ~53% of initial plantarflexion torque while sedentary mice lost ~58%. Data from the treadmill running and *in vivo* contractile tests suggest that simvastatin treatment did not impact physiological adaptations in fatigue resistance caused by endurance exercise training.

**Fig 2:**
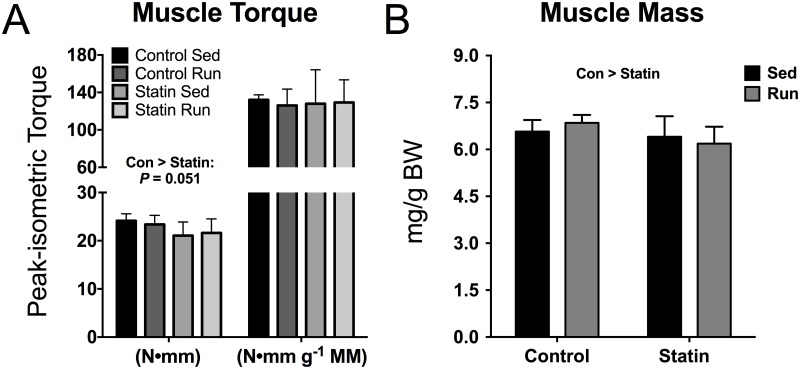
The effects of exercise training and simvastatin treatment on plantarflexor muscle torque and mass (n = 7–8 per exercise/treatment group). *A*: Peak isometric torque (left bars) and peak isometric torque normalized by plantarflexor muscle mass (right bars). Con > Statin indicates a strong trend for main effect of treatment, *P* = 0.051. *B*: Plantarflexor muscle masses normalized by grams of BW. Con > Statin indicates main effect of treatment, *P* = 0.039. The plantarflexor muscle mass represents the combined masses of the gastrocnemius, soleus, and plantaris muscles.

Statin users may report muscle weakness as a side effect to statin treatment [34]. There was a strong trend for lower torque in the plantarflexors of simvastatin treated mice compared to controls (*P* = 0.051, Fig 2A). However, the combined muscle mass of the plantarflexor (gastrocnemius, plantaris, soleus) muscles was ~6% lower in the simvastatin treated mice compared to control mice (*P* = 0.039, Fig 2B), and when ankle plantarflexion torque was normalized by muscle mass, no significant interaction or main effects were detected (*P* ≥ 0.62, Fig 2A).

### Gene expression

**Fig 3:**
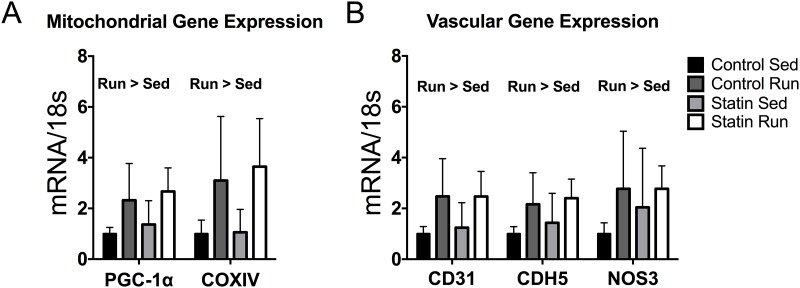
The effects of exercise training and simvastatin treatment on mitochondrial and vascular gene expressions (n = 7–8 per exercise/treatment group). *A*: Fold change relative to control sedentary mice in expression of genes involved in mitochondrial biogenesis. Run > Sed indicates main effect of training, all *P* < 0.002. *B*: Fold change relative to control sedentary mice in expression of genes involved in vascular signaling. Run > Sed indicates main effect of training, all *P* ≤ 0.046.

To determine the effects of simvastatin treatment on cellular level mitochondrial adaptations, we first examined *PGC-1α* and *COXIV* gene expression, which are reliable and well-established markers of mitochondrial biogenesis [17]. Trained mice had an ~2-fold greater *PGC-1α* expression (*P* < 0.003, Fig 3A) and ~3-fold greater *COXIV* expression compared to sedentary mice, independent of simvastatin treatment (*P* < 0.001, Fig 3A). We then proceeded to assess vascular gene responses to training and simvastatin treatment, as adaptations to the vascular network would be required to meet the demands of a larger mitochondria network. There was no effect of simvastatin treatment on vascular gene responses to endurance exercise training, and trained mice had a greater *CDH5* (*P <* 0.007, Fig 3B), *CD31* (*P* < 0.002, Fig 3B), and *NOS3* (*P* = 0.046, Fig 3B) expression compared to sedentary mice.

### Mitochondrial assays

To determine if greater *PGC1α* and *COXIV* gene expression was associated with greater mitochondrial content, we assessed several mitochondrial enzyme activities, including CS, which is strongly correlated to mitochondrial content [35], and β-HAD, which is an important enzyme for fatty acid oxidation. CS and β-HAD enzyme activities were ~11% and ~21% higher, respectively, in the trained mice compared to sedentary, independent of simvastatin treatment (*P* ≤ 0.028, 1). The gene and mitochondrial enzyme activity data did not support the hypothesis that a 6-week simvastatin regimen attenuates skeletal muscle adaptations to endurance exercise training. However, these tests do not reflect the quality or function of the mitochondria, which others have reported is negatively affected by statin treatment [16]. To determine mitochondrial function, we evaluated mitochondrial respiration rates from isolated mitochondria. There was no effect of training or simvastatin treatment on state 4, state 3, or state 3 uncoupled respiration of isolated mitochondria (*P* ≥ 0.30, Table 1).

**Table 1:** Mitochondrial content and function.

	Control	Control	Statin	Statin	Treatment	Exercise	Interaction
	Sedentary	Run	Sedentary	Run	*P value*	*P value*	*P value*
**Mitochondrial enzyme activities**							
CS	0.86 ± 0.056	0.95 ± 0.20*	0.87 ± 0.16	0.92 ± 0.075*	0.244	0.028	0.209
β-HAD	76.2 ± 14.7	95.1 ± 22.2*	73.0 ± 17.0	82.9 ± 10.6*	0.090	0.011	0.217
**Isolated mitochondrial respiration**							
Leak (state 4)	10.4 ± 2.86	11.9 ± 2.61*	10.5 ± 1.97	8.5 ± 2.40	0.805	0.374	0.822
Coupled (state 3)	44.2 ± 11.7	49.9 ± 14.1*	49.1 ± 4.87	50.1 ± 13.6	0.973	0.821	0.705
State 3 Uncoupled	80.7 ± 19.0	71.5 ± 27.5*	67.6 ± 4.62	72.0 ± 19.6	0.295	0.986	0.827

Note: Data are presented as mean ± SD. Mitochondrial enzyme activities are expressed as μmol/min/μg protein. Isolated mitochondrial respiration rates are expressed as nmol/min/μg and all rates were normalized by the citrate synthase activities of each fraction in order to account for differences in mitochondrial content between isolated mitochondrial fractions. CS: citrate synthase, β-HAD: β-hydroxy acyl-CoA dehydrogenase, SDH: succinate dehydrogenase.

*indicates main effect of training.

### Autophagy-related protein content

**Fig 4:**
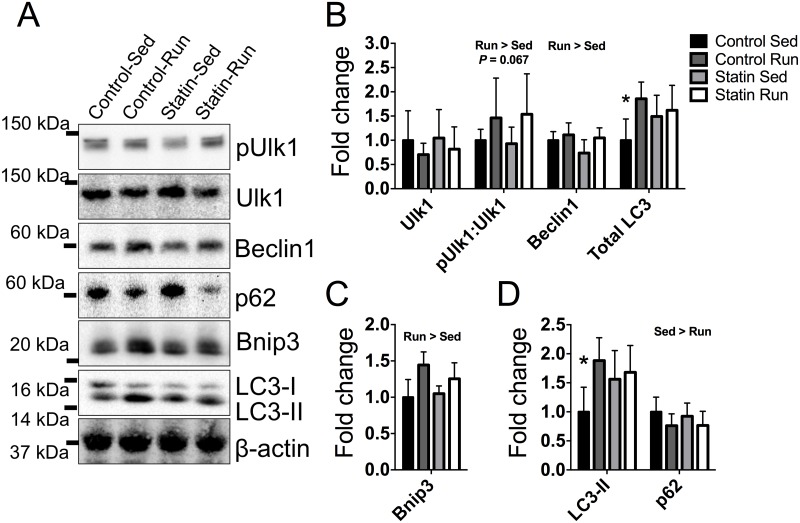
The effects of exercise training and simvastatin treatment on markers of autophagy. *A*: Immunoblot images of Ulk1, p-Ulk1:Ulk1, Beclin1, p62, Bnip3, LC3-I, and LC3-II in plantaris muscle. β-actin was used as a loading control. *B—C*: Quantitative and statistical analysis of data in *A* (sed n = 8; sed+statin n = 7; run n = 5; run+statin n = 8). Run > Sed indicates main effect of training, all *P* ≤ 0.049. * indicates significantly different from control run, statin sedentary and statin run, all *P* ≤ 0.046.

The proper regulation of mitochondrial quantity and quality involves both the addition of newly synthesized mitochondria to the reticulum via mitochondrial biogenesis, and the structural separation and disposal of damaged mitochondrial fragments via fission and autophagy. Autophagy is a cellular recycling process that identifies, removes, and degrades damaged or dysfunctional cellular components such as mitochondria that have been enveloped by a double-membrane structure called an autophagosome. We examined the impact of simvastatin treatment on autophagy because previous groups have reported that exercise training increases basal autophagy and that sufficient autophagy is required for exercise training-induced adaptations. Activation of unc-51 like kinase 1 (Ulk1, also referred to as Atg1) by AMPK phosphorylation of serine 555 is a critical step in the early induction of autophagy. There was no significant interaction or group effects for total Ulk1 protein content (*P* ≥ 0.201, Fig 4B); however, there was a strong trend for greater activation of Ulk1 (pUlk1:Ulk1) in exercise trained mice compared to sedentary mice, independent of simvastatin treatment (*P* = 0.067, Fig 4B). Beclin1 (or Atg6), which contributes to the formation of autophagosomes upon Ulk1 activation, was ~22% higher in trained mice compared to sedentary mice, independent of simvastatin treatment (*P* = 0.037, Fig 4B). There was a significant interaction between training and treatment for microtubule-associated proteins 1A/1B light chain 3A (LC3, or Atg8), which is a protein necessary for autophagosome elongation (*P* = 0.046, Fig 4B). Total LC3 protein content was between 49% and 85% greater in trained control mice, trained simvastatin mice, and sedentary simvastatin mice compared to sedentary control mice. BCL2/adenovirus E1B 19 kDa protein-interacting protein 3 (Bnip3), which is a mitochondria-specific receptor responsible for tagging the mitochondria for degradation, was 27% greater in trained mice compared to sedentary mice, independent of simvastatin treatment (~27%, *P* < 0.001, Fig 4C).

Autophagy flux describes a measure of the degradation activity of autophagy. Autophagy flux can be characterized by measuring the conjugated form of LC3, LC3-II, as well as the relative degradation of polyubiquitin-binding protein p62/SQSTM1 (p62). There was a significant interaction between exercise training and treatment for LC3-II protein content (*P* = 0.022), which, upon further analysis, revealed LC3-II protein content was between 56% and 88% greater in trained control mice, trained simvastatin mice, and sedentary simvastatin mice compared to sedentary control mice (Fig 4D). A relative reduction in p62 protein content is another indicator of autophagy flux, as p62 is degraded following delivery of the autophagosome to the lysosome. p62 protein content was ~23% less in trained mice compared to sedentary mice, independent of simvastatin treatment (*P* = 0.049, Fig 4D).

**Fig 5:**
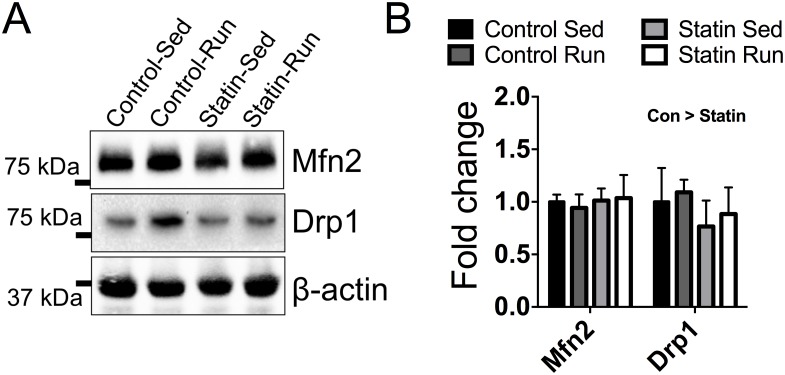
The impact of simvastatin treatment on mitochondrial fusion and fission. *A*: Immunoblot images of mitofusin 2 (Mfn2), and Drp1. β-actin was probed as a loading control. *B*: Quantitative and statistical analysis of data in *A* (sed n = 8; sed+statin n = 7; run n = 5; run+statin n = 8). Con > Statin indicates a main effect of treatment, *P* = 0.040.

To determine the extent to which training and/or simvastatin treatment impacted mitochondrial dynamics, we measured protein contents related to mitochondrial fission and fusion, two processes necessary for mitochondrial maintenance. There were no significant interactions or main effects for mitochondrial fission protein mitofusin 2 (*P* ≥ 0.392, Fig 5B). Dynamin-related protein 1 (Drp1), is a protein necessary for mitochondrial fission during autophagy [36], and was ~24% lower in simvastatin treated mice compared to control mice, independent of exercise training (*P = *0.040, Fig 5B).

## Discussion

This study systematically evaluated the impact of simvastatin treatment on chronic exercise training adaptations spanning from the whole body to the cellular level. The rationale for this study was a dearth of information regarding the impact of statins on tissue-specific cellular adaptations to exercise. We hypothesized that statins would attenuate skeletal muscle adaptations to endurance exercise training based on an accumulating body of literature reporting statin-induced skeletal muscle problems including myalgia and weakness [4, 37] as well as suggestions of mitochondrial dysfunction [12, 14–16, 18] and blunted training-induced cardiovascular and skeletal muscle adaptations in statin-treated humans [13]. Our hypothesis was not supported as there was minimal evidence of adverse effects from simvastatin treatment at the conclusion of our 6-week study. Overall, simvastatin treated mice were capable of endurance exercise training adaptations comparable to untreated mice; however, we did observe a strong trend for lower muscle torque as well as modest differences in basal autophagy between untreated and statin-treated mice which are discussed below.

Muscle weakness is a well-documented complaint among statin users [34, 38]. However, few studies have directly assessed muscle weakness in statin users, and the studies that are available have produced mixed results [5–10, 37, 39, 40]. For example, Scott et al. reported decreased leg strength in 179 older statin users [37], while Parker et al. did not detect statin-induced arm or leg muscle weakness in a study including 203 statin users [10]. Our *in vivo* contractility tests are a truly maximal assessment of muscle torque as muscle contractions are evoked by direct nerve stimulation in a quantifiable manner that is independent of subject motivation. We observed a strong trend for lower peak isometric torque of the ankle plantarflexors in simvastatin treated mice (Fig 2B); however, this trend disappeared when torque was normalized by muscle mass, indicating that smaller muscles (Fig 2A) were underlying the trend in muscle weakness. A shift in the balance between muscle protein synthesis and degradation toward protein degradation results in a reduction in muscle size, i.e. muscle atrophy. Indeed, previous studies using cell culture and rodent models have found that statin treatment leads to muscle atrophy through statin-mediated suppression of AKT/mTOR signaling, activation of caspases, and upregulation of ubiquitin proteasome system E3-ligases (atrogin-1 and MuRF1) [41, 42]. Importantly, suppression of AKT phosphorylation and upregulation of Atrogin-1 have also been observed in human statin users who exhibit signs of muscle myopathies [43, 44]. Overall, our results are in line with previous research suggesting that statins could alter protein synthesis signaling pathways and induce muscle atrophy, but further research is needed in human statin users to explore whether these alterations are associated with any appreciable effects in the skeletal muscle such as muscle weakness.

**Fig 6:**
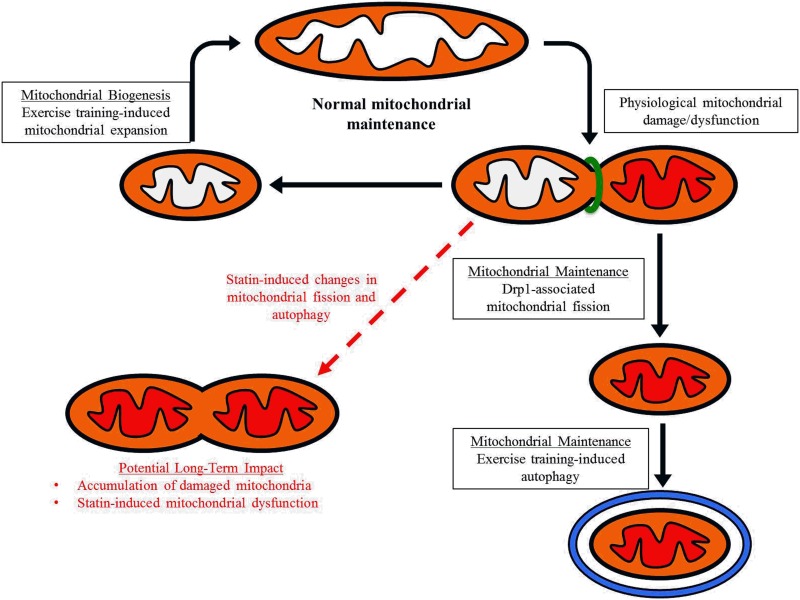
Exercise training-induced effects on mitochondrial biogenesis and maintenance, and potential contraindicative effects of statin use. Normal mitochondrial maintenance (black arrows) is theoretically characterized by the balance between the addition of healthy mitochondria (mitochondria with white fill) via mitochondrial biogenesis and removal of damaged mitochondria (mitochondrial with red fill) via autophagy. Herein, short-term statin use was associated with a decrease in mitochondrial fission protein Drp1 and altered autophagy-related protein LC3 content, which may contribute to disrupted mitochondrial maintenance. Long-term disruption in the mitochondrial maintenance (red dashed arrow) may lead to accumulation of damaged mitochondria. More directed research is necessary to understand the potential long-term impact of statin use on mitochondrial fission, autophagy, and function.

Autophagy is a cellular recycling process that plays an important role in skeletal muscle mitochondrial maintenance. Basal levels of autophagy increase following exercise training, and this is characterized by formation of autophagosomes that engulf and dispose of specific cellular structures such as mitochondria that have been targeted for degradation (Fig 6). In accordance with previous findings [45], we found exercise trained mice had greater levels of the autophagy-related proteins necessary for autophagosome formation and expansion (Fig 4A). However, we also found that simvastatin treated mice had lower levels of Drp1 (Fig 5B), which is an essential protein for mitochondrial fission during autophagy. Mitochondrial fission is the structural separation of mitochondria from the mitochondrial network, a critical step for the successful removal of damaged or dysfunctional mitochondria during autophagy. Long-term reductions in mitochondrial fission as the result of a chronic statin use could potentially have negative consequences on the function of the mitochondrial network and basal autophagy (Fig 6). In line with this hypothesis, IIkeda et al. used a conditional Drp1 knockout mouse and shRNA to downregulate Drp1 *in vivo* and *in vitro*, respectively, and demonstrated that Drp1 was critical for mitochondrial function (i.e., ATP production) and autophagosome assembly [46]. The short-term design of this current study may have prevented us from detecting the long-term consequences of insufficient Drp1-mediated mitochondrial fission on mitochondrial function, but our LC3-II data can provide some insight into the impact of simvastatin on autophagosome assembly.

Autophagosome assembly can be measured via LC3-II protein content accumulation and used as an indicator of autophagy flux. While accumulation of LC3-II protein content on an immunoblot (autophagosome accumulation) can represent ongoing autophagy flux, it is also used as a marker to confirm successful autophagy inhibition in the presence of known pharmacological autophagy inhibitors [47]. In the current study, accumulation of LC3-II in untreated exercise trained control mice can be interpreted as autophagy flux considering the previous work by Lira et al. [45] and considering our own analysis of pUlk1, Beclin1, Bnip3, and p62 protein contents (Fig 4A). However, the LC3-II protein content in simvastatin treated mice are harder to interpret given that LC3-II accumulation is present in both sedentary and exercise trained mice (Fig 4C). When the LC3-II data is interpreted along with the lower Drp1 protein levels in simvastatin treated mice, one could reasonably consider that simvastatin treatment potentially results in impairment in autophagosome elongation and maturation (i.e., autophagy inhibition). We are not the first to report LC3-II accumulation with simvastatin treatment [48], but our comprehensive autophagy-related protein analysis suggests more research is necessary in order to determine the long-term benefit/detriments of statins on skeletal muscle autophagy, especially when considering the efficacy of statin treatment alongside comorbidities such as muscular dystrophy, aging, and cardiovascular disease.

Increased skeletal muscle mitochondrial content is a hallmark adaptation to exercise training. Following repeated muscular contractions, mitochondrial biogenesis signaling pathways are activated resulting in overall expansion of the mitochondrial network within the muscle [49]. Recently, Mikus et al. [13] demonstrated that the typical exercise-induced improvements in mitochondrial content, as assessed by citrate synthase activity, are blunted in individuals also consuming statins. In contrast, when we performed the same citrate synthase assay as a proxy for mitochondrial content we did not find blunted adaptations in trained statin-treated mice. The reason why our results do not agree with Mikus et al. could be due to differences in experimental approach, specifically, we used a murine model and not human participants to test our hypothesis. However, the strength of this study is that we used several additional indicators of mitochondrial content including physiological changes in fatigue resistance (i.e. treadmill running, contractile fatigue test, Fig 1), gene expression, and autophagy-related protein contents, which supported our primary conclusion that training adaptations in mitochondrial content are attainable with simvastatin treatment.

Statin treatment has been proposed to cause skeletal muscle mitochondrial dysfunction [11, 12, 15, 16], which may underlie some muscular myopathies, such as weakness or fatigue, reported by statin users. Herein we evaluated mitochondrial function primarily through isolated mitochondrial respiration, but also indirectly through skeletal muscle and whole body fatigue protocols. Interestingly, in contrast to the previous studies showing mitochondrial dysfunction with statin treatment [12, 16, 18, 50, 51], we found no differences in mitochondrial function between control and simvastatin treated mice. The discrepancy between previously published work and ours may be due to a couple of reasons. First, several studies reporting statin-induced mitochondrial dysfunction relied on cell culture systems involving high non-physiological doses (1000 fold higher) of statin prior to measuring mitochondrial function [16, 50, 52]. Second, in contrast to other studies with longer treatment regimens [12, 18, 51], we found that 6 weeks of simvastatin treatment did not result in mitochondrial dysfunction. Considering our Drp1 and LC3-II data, the 6-weeks of simvastatin treatment used in this study could have been insufficient to observe an accumulation of dysfunctional mitochondria required to detect significant reduction in mitochondrial respiration (Fig 6). Nonetheless, our results indicate that a 6-week treatment of simvastatin does not result in skeletal muscle mitochondrial dysfunction.

An important element of this study was choosing the appropriate simvastatin dose to administer to the mice. Previous animal studies have used a wide range of simvastatin doses, ranging anywhere from 5 and 300 mg/kg [21, 53–61]. However, we wanted to select a simvastatin dose that would be both clinically relevant and physiologically appropriate. In order for the simvastatin dose to be clinically relevant, we chose to give the mice a simvastatin dose that would be the human equivalent of 100 mg/kg, which is similar to the highest recommended human simvastatin dose of 80 mg/kg. In order to achieve a physiologically appropriate dose, we converted the human simvastatin dose of 100 mg/kg to the target murine dose of 20 mg/kg using previously published human to animal dose translation calculations [20]. At the end of this study, the sedentary mice received a simvastatin dose similar to our target dose (22.1 ± 2.8 mg/kg), while the trained mice consumed more water and consequently received a simvastatin dose greater than the target dose (34.8 ± 5.4 mg/kg). We do not believe, however, that the dosage difference between the trained and untrained mice affected the results of the experiments. For example, statistically, there was no instance in any of the outcome measurements where the statin-treated trained mice were different from the untreated trained mice, or where the statin-treated trained mice were different from the statin-treated sedentary mice. In addition, the trained mice receiving the high simvastatin dose did not display any signs of negative side effects. Overall, while future experimental designs should account for the difference in water consumed, it is unlikely that the difference in statin dosing between groups impacted the outcomes of this study.

## Conclusion

Collectively, our results indicated that exercise training adaptations at the whole-body and muscle-cell level are attainable with simvastatin treatment. While this may be good news for statin users also engaging in exercise, more research is needed to investigate potential contraindicative effects of long-term statin use in skeletal muscle, including their effects on muscle protein synthesis, degradation, and autophagy (Fig 6).

## References

[1] Mozaffarian D, Benjamin EJ, Go AS, Arnett DK, Blaha MJ, Cushman M, et al Heart disease and stroke statistics—2015 update: a report from the American Heart Association. Circulation. 2015;131(4):e29–322. 10.1161/CIR.0000000000000152 25520374

[2] Taylor FC, Huffman M, Ebrahim S. Statin therapy for primary prevention of cardiovascular disease. JAMA. 2013;310(22):2451–2. 10.1001/jama.2013.281348 24276813

[3] Shiroma EJ, Lee IM. Physical activity and cardiovascular health: lessons learned from epidemiological studies across age, gender, and race/ethnicity. Circulation. 2010;122(7):743–52. 10.1161/CIRCULATIONAHA.109.914721 20713909

[4] Ganga HV, Slim HB, Thompson PD. A systematic review of statin-induced muscle problems in clinical trials. Am Heart J. 2014;168(1):6–15. 10.1016/j.ahj.2014.03.019 24952854

[5] Whitehead NP. Enhanced autophagy as a potential mechanism for the improved physiological function by simvastatin in muscular dystrophy. Autophagy. 2016;12(4):705–6. 10.1080/15548627.2016.1144005 26890413 PMC4835971

[6] Panza GA, Taylor BA, Dada MR, Thompson PD. Changes in muscle strength in individuals with statin-induced myopathy: A summary of 3 investigations. J Clin Lipidol. 2015;9(3):351–6. 10.1016/j.jacl.2015.01.004 26073393

[7] Rengo JL, Callahan DM, Savage PD, Ades PA, Toth MJ. Skeletal muscle ultrastructure and function in statin-tolerant individuals. Muscle Nerve. 2016;53(2):242–51. 10.1002/mus.24722 26059690 PMC4673037

[8] Davis ME, Korn MA, Gumucio JP, Harning JA, Saripalli AL, Bedi A, et al Simvastatin reduces fibrosis and protects against muscle weakness after massive rotator cuff tear. J Shoulder Elbow Surg. 2015;24(2):280–7. 10.1016/j.jse.2014.06.048 25213828 PMC4291297

[9] Ballard KD, Parker BA, Capizzi JA, Grimaldi AS, Clarkson PM, Cole SM, et al Increases in creatine kinase with atorvastatin treatment are not associated with decreases in muscular performance. Atherosclerosis. 2013;230(1):121–4. 10.1016/j.atherosclerosis.2013.07.001 23958263 PMC3779874

[10] Ba Parker, Ja Capizzi, Grimaldi AS, Clarkson PM, Cole SM, Keadle J, et al Effect of statins on skeletal muscle function. Circulation. 2013;127(1):96–103. 10.1161/CIRCULATIONAHA.112.136101 23183941 PMC4450764

[11] Bouitbir J, Charles AL, Rasseneur L, Dufour S, Piquard F, Geny B, et al Atorvastatin treatment reduces exercise capacities in rats: involvement of mitochondrial impairments and oxidative stress. J Appl Physiol (1985). 2011;111(5):1477–83. 21852406 10.1152/japplphysiol.00107.2011

[12] Muraki A, Miyashita K, Mitsuishi M, Tamaki M, Tanaka K, Itoh H. Coenzyme Q10 reverses mitochondrial dysfunction in atorvastatin-treated mice and increases exercise endurance. J Appl Physiol. 2012;113(3):479–86. 10.1152/japplphysiol.01362.2011 22653988

[13] Mikus CR, Boyle LJ, Borengasser SJ, Oberlin DJ, Naples SP, Fletcher J, et al Simvastatin impairs exercise training adaptations. J Am Coll Cardiol. 2013;62(8):709–14. 10.1016/j.jacc.2013.02.074 23583255 PMC3745788

[14] Sirvent P, Fabre O, Bordenave S, Hillaire-Buys D, Raynaud De Mauverger E, Lacampagne A, et al Muscle mitochondrial metabolism and calcium signaling impairment in patients treated with statins. Toxicol Appl Pharmacol. 2012;259(2):263–8. 10.1016/j.taap.2012.01.008 22269104

[15] Wu JS, Buettner C, Smithline H, Ngo LH, Greenman RL. Evaluation of skeletal muscle during calf exercise by 31-phosphorus magnetic resonance spectroscopy in patients on statin medications. Muscle Nerve. 2011;43(1):76–81. 10.1002/mus.21847 21171098 PMC3332539

[16] Kwak H-B, Thalacker-Mercer A, Anderson EJ, Lin C-T, Kane DA, Lee N-S, et al Simvastatin impairs ADP-stimulated respiration and increases mitochondrial oxidative stress in primary human skeletal myotubes. Free radical biology & medicine. 2012;52(1):198–207. 22080086 10.1016/j.freeradbiomed.2011.10.449 PMC3313473

[17] Wu Z, Puigserver P, Andersson U, Zhang C, Adelmant G, Mootha V, et al Mechanisms controlling mitochondrial biogenesis and respiration through the thermogenic coactivator PGC-1. Cell. 1999;98(1):115–24. 10.1016/S0092-8674(00)80611-X 10412986

[18] Päivä H, Thelen KM, Van Coster R, Smet J, De Paepe B, Mattila KM, et al High-dose statins and skeletal muscle metabolism in humans: A randomized, controlled trial. Clin Pharmacol Ther. 2005;78(1):60–8. 10.1016/j.clpt.2005.03.006 16003294

[19] Gu Q, Paulose-Ram R, Burt VL, Kit BK. Prescription cholesterol-lowering medication use in adults aged 40 and over: United States, 2003–2012. NCHS Data Brief. 2014;(177):1–8. 25536410

[20] Reagan-Shaw S, Nihal M, Ahmad N. Dose translation from animal to human studies revisited. The FASEB journal: official publication of the Federation of American Societies for Experimental Biology. 2008;22(3):659–61. 17942826 10.1096/fj.07-9574LSF

[21] Whitehead NP, Kim MJ, Bible KL, Adams ME, Froehner SC. A new therapeutic effect of simvastatin revealed by functional improvement in muscular dystrophy. Proc Natl Acad Sci U S A. 2015;112(41):12864–9. 10.1073/pnas.1509536112 26417069 PMC4611601

[22] Fox JG, Barthold SW, Davisson MT, Newcomer CE, Quimby FW, Smith AL. The Mouse in Biomedical Research Volume 3: Normative Biology, Husbandry, and Models. 2nd ed Burlington, MA: Elsevier, AP; 2007.

[23] Call JA, McKeehen JN, Novotny SA, Lowe DA. Progressive resistance voluntary wheel running in the mdx mouse. Muscle Nerve. 2010;42(6):871–80. 10.1002/mus.21764 21104862 PMC3392646

[24] Li P, Waters RE, Redfern SI, Zhang M, Mao L, Annex BH, et al Oxidative phenotype protects myofibers from pathological insults induced by chronic heart failure in mice. Am J Pathol. 2007;170(2):599–608. 10.2353/ajpath.2007.060505 17255328 PMC1851852

[25] Okutsu M, Call JA, Lira VA, Zhang M, Donet JA, French BA, et al Extracellular superoxide dismutase ameliorates skeletal muscle abnormalities, cachexia, and exercise intolerance in mice with congestive heart failure. Circ Heart Fail. 2014;7(3):519–30. 10.1161/CIRCHEARTFAILURE.113.000841 24523418 PMC4080303

[26] Baltgalvis KA, Call JA, Nikas JB, Lowe DA. Effects of prednisolone on skeletal muscle contractility in mdx mice. Muscle Nerve. 2009;40(3):443–54. 10.1002/mus.21327 19618428 PMC2879072

[27] Call JA, Ervasti JM, Lowe DA. TAT-muUtrophin mitigates the pathophysiology of dystrophin and utrophin double-knockout mice. J Appl Physiol (1985). 2011;111(1):200–5. 21565990 10.1152/japplphysiol.00248.2011 PMC3137527

[28] Baltgalvis KA, Call JA, Cochrane GD, Laker RC, Yan Z, Lowe DA. Exercise training improves plantar flexor muscle function in mdx mice. Med Sci Sports Exerc. 2012;44(9):1671–9. 10.1249/MSS.0b013e31825703f0 22460476 PMC3470762

[29] Gorassini M, Eken T, Bennett DJ, Kiehn O, Hultborn H. Activity of hindlimb motor units during locomotion in the conscious rat. J Neurophysiol. 2000;83(4):2002–11. 10758110 10.1152/jn.2000.83.4.2002

[30] Jenkins NT, Landers RQ, Thakkar SR, Fan X, Brown MD, Prior SJ, et al Prior endurance exercise prevents postprandial lipaemia-induced increases in reactive oxygen species in circulating CD31+ cells. J Physiol. 2011;589(Pt 22):5539–53. 10.1113/jphysiol.2011.215277 21930598 PMC3240890

[31] Nichenko AS, Southern WM, Atuan M, Luan J, Peissig KB, Foltz SJ, et al Mitochondrial maintenance via autophagy contributes to functional skeletal muscle regeneration and remodeling. Am J Physiol Cell Physiol. 2016;311(2):C190–200. 10.1152/ajpcell.00066.2016 27281480

[32] Garcia-Cazarin ML, Snider NN, Andrade FH. Mitochondrial isolation from skeletal muscle. J Vis Exp. 2011;(49). 10.3791/2452 PMC3197291 21490576

[33] Call JA, Chain KH, Martin KS, Lira VA, Okutsu M, Zhang M, et al Enhanced skeletal muscle expression of extracellular superoxide dismutase mitigates streptozotocin-induced diabetic cardiomyopathy by reducing oxidative stress and aberrant cell signaling. Circ Heart Fail. 2015;8(1):188–97. 10.1161/CIRCHEARTFAILURE.114.001540 25504759 PMC4445759

[34] Krishnan GM, Thompson PD. The effects of statins on skeletal muscle strength and exercise performance. Curr Opin Lipidol. 2010;21(4):324–8. 10.1097/MOL.0b013e32833c1edf 20581676

[35] Larsen S, Nielsen J, Hansen CN, Nielsen LB, Wibrand F, Stride N, et al Biomarkers of mitochondrial content in skeletal muscle of healthy young human subjects. J Physiol. 2012;590(14):3349–60. 10.1113/jphysiol.2012.230185 22586215 PMC3459047

[36] Otera H, Ishihara N, Mihara K. New insights into the function and regulation of mitochondrial fission. Biochim Biophys Acta. 2013;1833(5):1256–68. 10.1016/j.bbamcr.2013.02.002 23434681

[37] Scott D, Blizzard L, Fell J, Jones G. Statin therapy, muscle function and falls risk in community-dwelling older adults. QJM. 2009;102(9):625–33. 10.1093/qjmed/hcp093 19633029

[38] Bruckert E, Hayem G, Dejager S, Yau C, Bégaud B. Mild to moderate muscular symptoms with high-dosage statin therapy in hyperlipidemic patients—The PRIMO study. Cardiovasc Drugs Ther. 2005;19(6):403–14. 10.1007/s10557-005-5686-z 16453090

[39] Ashfield TA, Syddall HE, Martin HJ, Dennison EM, Cooper C, Aihie Sayer A. Grip strength and cardiovascular drug use in older people: findings from the Hertfordshire Cohort Study. Age Ageing. 2010;39(2):185–91. 10.1093/ageing/afp203 20019032 PMC3546312

[40] Traustadottir T, Stock AA, Harman SM. High-dose statin use does not impair aerobic capacity or skeletal muscle function in older adults. Age. 2008;30(4):283–91. 10.1007/s11357-008-9070-3 19424852 PMC2585641

[41] Goodman CA, Pol D, Zacharewicz E, Lee-Young RS, Snow RJ, Russell AP, et al Statin-Induced Increases in Atrophy Gene Expression Occur Independently of Changes in PGC1alpha Protein and Mitochondrial Content. PLoS ONE. 2015;10(5):e0128398 10.1371/journal.pone.0128398 26020641 PMC4447258

[42] Bonifacio A, Sanvee GM, Bouitbir J, Krahenbuhl S. The AKT/mTOR signaling pathway plays a key role in statin-induced myotoxicity. Biochim Biophys Acta. 2015;1853(8):1841–9. 10.1016/j.bbamcr.2015.04.010 25913013

[43] Mallinson JE, Constantin-Teodosiu D, Sidaway J, Westwood FR, Greenhaff PL. Blunted Akt/FOXO signalling and activation of genes controlling atrophy and fuel use in statin myopathy. J Physiol. 2009;587(1):219–30. 10.1113/jphysiol.2008.164699 19001041 PMC2670035

[44] Hanai J, Cao P, Tanksale P, Imamura S, Koshimizu E, Zhao J, et al The muscle-specific ubiquitin ligase atrogin-1/MAFbx mediates statin-induced muscle toxicity. J Clin Invest. 2007;117(12):3940–51. 17992259 10.1172/JCI32741 PMC2066198

[45] Lira VA, Okutsu M, Zhang M, Greene NP, Laker RC, Breen DS, et al Autophagy is required for exercise training-induced skeletal muscle adaptation and improvement of physical performance. FASEB J. 2013;27(10):4184–93. 10.1096/fj.13-228486 23825228 PMC4046188

[46] Ikeda Y, Shirakabe A, Maejima Y, Zhai P, Sciarretta S, Toli J, et al Endogenous Drp1 mediates mitochondrial autophagy and protects the heart against energy stress. Circ Res. 2015;116(2):264–78. 10.1161/CIRCRESAHA.116.303356 25332205

[47] Mizushima N, Yoshimori T, Levine B. Methods in mammalian autophagy research. Cell. 2010;140(3):313–26. 10.1016/j.cell.2010.01.028 20144757 PMC2852113

[48] Whitehead NP, Kim MJ, Bible KL, Adams ME, Froehner SC. A new therapeutic effect of simvastatin revealed by functional improvement in muscular dystrophy. Proceedings of the National Academy of Sciences. 2015;112(41):12864–9. 10.1073/pnas.1509536112 PMC4611601 26417069

[49] Hood DA. Invited Review: contractile activity-induced mitochondrial biogenesis in skeletal muscle. J Appl Physiol (1985). 2001;90(3):1137–57. 11181630 10.1152/jappl.2001.90.3.1137

[50] Bouitbir J, Daussin F, Charles AL, Rasseneur L, Dufour S, Richard R, et al Mitochondria of trained skeletal muscle are protected from deleterious effects of statins. Muscle Nerve. 2012;46(3):367–73. 10.1002/mus.23309 22907227

[51] Schick BA, Laaksonen R, Frohlich JJ, Paiva H, Lehtimaki T, Humphries KH, et al Decreased skeletal muscle mitochondrial DNA in patients treated with high-dose simvastatin. Clin Pharmacol Ther. 2007;81(5):650–3. Epub 2007/03/03. 10.1038/sj.clpt.6100124 17329991

[52] Bjorkhem-Bergman L, Lindh JD, Bergman P. What is a relevant statin concentration in cell experiments claiming pleiotropic effects? Br J Clin Pharmacol. 2011;72(1):164–5. 10.1111/j.1365-2125.2011.03907.x 21223360 PMC3141200

[53] Bea F. Simvastatin Promotes Atherosclerotic Plaque Stability in ApoE-Deficient Mice Independently of Lipid Lowering. Arterioscler Thromb Vasc Biol. 2002;22(11):1832–7. 12426212 10.1161/01.atv.0000036081.01231.16

[54] Bea F, Blessing E, Shelley MI, Shultz JM, Rosenfeld ME. Simvastatin inhibits expression of tissue factor in advanced atherosclerotic lesions of apolipoprotein E deficient mice independently of lipid lowering: potential role of simvastatin-mediated inhibition of Egr-1 expression and activation. Atherosclerosis. 2003;167(2):187–94. 12818400 10.1016/s0021-9150(02)00387-8

[55] Choudhury RP, Carrelli AL, Stern JD, Chereshnev I, Soccio R, Elmalem VI, et al Effects of Simvastatin on Plasma Lipoproteins and Response to Arterial Injury in Wild-Type and Apolipoprotein-E-Deficient Mice. J Vasc Res. 2004;41(1):75–83. 10.1159/000076436 14752252

[56] Leung BP, Sattar N, Crilly A, Prach M, McCarey DW, Payne H, et al A Novel Anti-Inflammatory Role for Simvastatin in Inflammatory Arthritis. The Journal of Immunology. 2003;170(3):1524–30. 12538717 10.4049/jimmunol.170.3.1524

[57] Ra Miller, Harrison DE, Astle CM, Baur JA, Boyd AR, de Cabo R, et al Rapamycin, But Not Resveratrol or Simvastatin, Extends Life Span of Genetically Heterogeneous Mice. The Journals of Gerontology Series A: Biological Sciences and Medical Sciences. 2011;66A(2):191–201. 10.1093/gerona/glq178 PMC3021372 20974732

[58] Palmer G, Chobaz V, Talabot-Ayer D, Taylor S, So A, Gabay C, et al Assessment of the efficacy of different statins in murine collagen-induced arthritis. Arthritis Rheum. 2004;50(12):4051–9. 10.1002/art.20673 15593180

[59] Skoglund RN, Forslund C, Aspenberg PER. Simvastatin Improves Fracture Healing in Mice. 2004;17(11):2004–8. 10.1359/jbmr.2002.17.11.2004 12412808

[60] Tong XK, Lecrux C, Rosa-Neto P, Hamel E. Age-dependent rescue by simvastatin of Alzheimer's disease cerebrovascular and memory deficits. J Neurosci. 2012;32(14):4705–15. 10.1523/JNEUROSCI.0169-12.2012 22492027 PMC6620905

[61] Wang YX, Martin-McNulty B, Huw LY, da Cunha V, Post J, Hinchman J, et al Anti-atherosclerotic effect of simvastatin depends on the presence of apolipoprotein E. Atherosclerosis. 2002;162(1):23–31. 11947894 10.1016/s0021-9150(01)00678-5

